# Effects of Hydroxyapatite Pretreated with Two Sodium Fluoride Concentrations on Early Bone Healing in Beagle Dogs

**DOI:** 10.3390/ijms27219414

**Published:** 2026-10-22

**Authors:** Yoshino Suzuki, Ujjal K. Bhawal, Aman Chowdhry, Takao Watanabe, Hiroyuki Okada, Kazunari Kimoto

**Affiliations:** 1 Department of Histology and Embryology, Nihon University Graduate School of Dentistry at Matsudo, Matsudo 271-8587, Japan; mayo23008@g.nihon-u.ac.jp; 2 Department of Physiology, Saveetha Medical College and Hospital, Saveetha Institute of Medical and Technical Sciences, Chennai 600077, India; 3 Research Institute of Oral Science, Nihon University School of Dentistry at Matsudo, Matsudo 271-8587, Japan; 4 Department of Oral Pathology, Faculty of Dentistry, Jamia Millia Islamia, New Delhi 110025, India; achowdhry@jmi.ac.in; 5 Japan Oral Medicine Organization, Tokyo 135-0033, Japan; 21616twatanabe@gmail.com; 6 Department of Histology, Nihon University School of Dentistry at Matsudo, Matsudo 271-8587, Japan; okada.hiroyuki@nihon-u.ac.jp; 7 School of Dentistry, Kanagawa Dental University, Yokosuka 238-8580, Japan; kzkimoto@gmail.com

**Keywords:** sodium fluoride, hydroxyapatite, bone healing, osteogenesis, Runx2, osteocalcin, beagle dog

## Abstract

Hydroxyapatite (HA) is widely used as an osteoconductive bone substitute, and fluoride can influence bone-forming cells in a concentration-dependent manner. This study examined how pretreating resorbable HA with low or high concentrations of sodium fluoride (NaF) affects early mandibular bone healing in beagle dogs. Twelve beagle dogs were used in the project, including three in a pilot study and nine in the main experiment. At each observation time point (Days 4, 7, and 14), one dog was assigned to each of the three HA-based treatment conditions (HA, HA + 0.2% NaF, or HA + 4% NaF). Four experimental defects in each dog received the same assigned treatment, while four contralateral defects were left unfilled as controls. The four defects were considered within-animal subsamples rather than independent biological replicates, and all treatment comparisons were descriptive and exploratory. Before implantation, HA was exposed to the designated NaF solution for 3 min and then washed with 100 mL of distilled water. Healing was evaluated by H&E, Azan, Masson’s trichrome and toluidine blue staining, immunohistochemistry, and quantitative real-time RT-PCR. Histological examination showed progressive tissue and matrix organization around the implanted material, with more evident new bone and osteoblast-associated changes in the low-NaF group at Days 7 and 14. Runx2 immunoreactivity was more prominent in the early healing phase, and osteocalcin was more prominent in the later healing phase. Osteocalcin mRNA gradually increased until Day 14, whereas Runx2 mRNA was maximally expressed at Day 7. Both responses were strongest with low-NaF-pretreated HA. High-NaF-pretreated HA retained osteogenic activity but did not produce a response of the same magnitude. These exploratory findings suggest that NaF pretreatment concentration may influence early HA-associated bone healing, warranting confirmation in studies with independent animal-level replication.

## 1. Introduction

Reconstruction of alveolar and maxillofacial bone defects remains an important challenge in oral surgery, implant dentistry, and regenerative dentistry. Synthetic calcium-phosphate materials are commonly used when an osteoconductive scaffold is needed, and hydroxyapatite (HA) has been studied extensively because its mineral composition resembles that of bone and it supports cell attachment, tissue ingrowth, and new bone formation [1,2,3]. The biological behavior of HA is not fixed, however, and can be influenced by its composition, crystallinity, resorbability, surface characteristics, and ionic substitutions [2,3].

Fluoride is readily incorporated into apatite structures and can alter both the physicochemical properties of HA and the local cellular environment at the bone-material interface. Earlier studies showed that crystalline fluorapatite-coated HA could be prepared successfully and support osteogenesis in a canine model [4,5]. Fluoride-treated bi-resorbable synthetic HA has also been evaluated in beagle mandibular defects, providing a basis for using HA as a local carrier of fluoride during bone healing [6]. In addition, fluorinated porcine HA has been reported to improve new-bone formation and bone-volume parameters compared with non-fluorinated HA in canine mandibular ridge defects [7]. More recent studies have extended these findings, showing that fluoride-substituted or fluorinated HA can support osteogenic activity and bone regeneration across different scaffold systems and experimental models [8,9,10,11].

The biological response to fluoride, however, depends strongly on concentration. Fluoride has long been recognized as exerting biphasic effects on bone and bone-forming cells [12,13]. NaF can promote proliferation, differentiation, alkaline phosphatase activity, mineralization and expression of osteogenic genes such as Runx2, Osterix, osteopontin and osteocalcin in osteoblasts at appropriate concentrations [14]. We also previously showed that low-level NaF can enhance cell migration, wound healing responses and osteogenic differentiation [15,16,17]. In contrast, excessive fluoride exposure can disturb cellular homeostasis and is associated with oxidative stress, apoptosis and impaired autophagy [18]. Recent work also suggests that the regenerative effects of fluoride-containing HA extend beyond direct effects on osteogenic cells and may involve changes in the local immune and vascular microenvironment [19,20,21].

Runx2 and osteocalcin provide complementary information about the progression of osteoblast differentiation. Runx2 is a key transcriptional regulator of osteoblast lineage commitment and early differentiation [22,23,24], whereas osteocalcin is more closely associated with differentiated osteoblasts and the mineralizing bone matrix. Examining these markers during the early healing period can therefore help distinguish early osteogenic commitment from later matrix maturation.

Although fluoride-modified HA has shown regenerative potential, relatively little is known about how the concentration used to pretreat resorbable HA shapes the very early molecular response in a large-animal mandibular defect model. It remains unclear whether a higher NaF pretreatment concentration necessarily produces a stronger osteogenic response or whether a lower concentration provides a more favorable local environment for healing.

The present study therefore investigated early bone healing associated with resorbable HA pretreated with two NaF concentrations in beagle dogs. Untreated HA, HA pretreated with 0.2% NaF, and HA pretreated with 4% NaF were compared with non-implanted control defects at Days 4, 7, and 14. Three-dimensional imaging and histological analyses were combined with immunohistochemical and quantitative real-time RT-PCR assessment of Runx2 and osteocalcin to characterize the temporal osteogenic response.

## 2. Results

### 2.1. Histological Changes During Early Bone Healing

**Figure 1:**
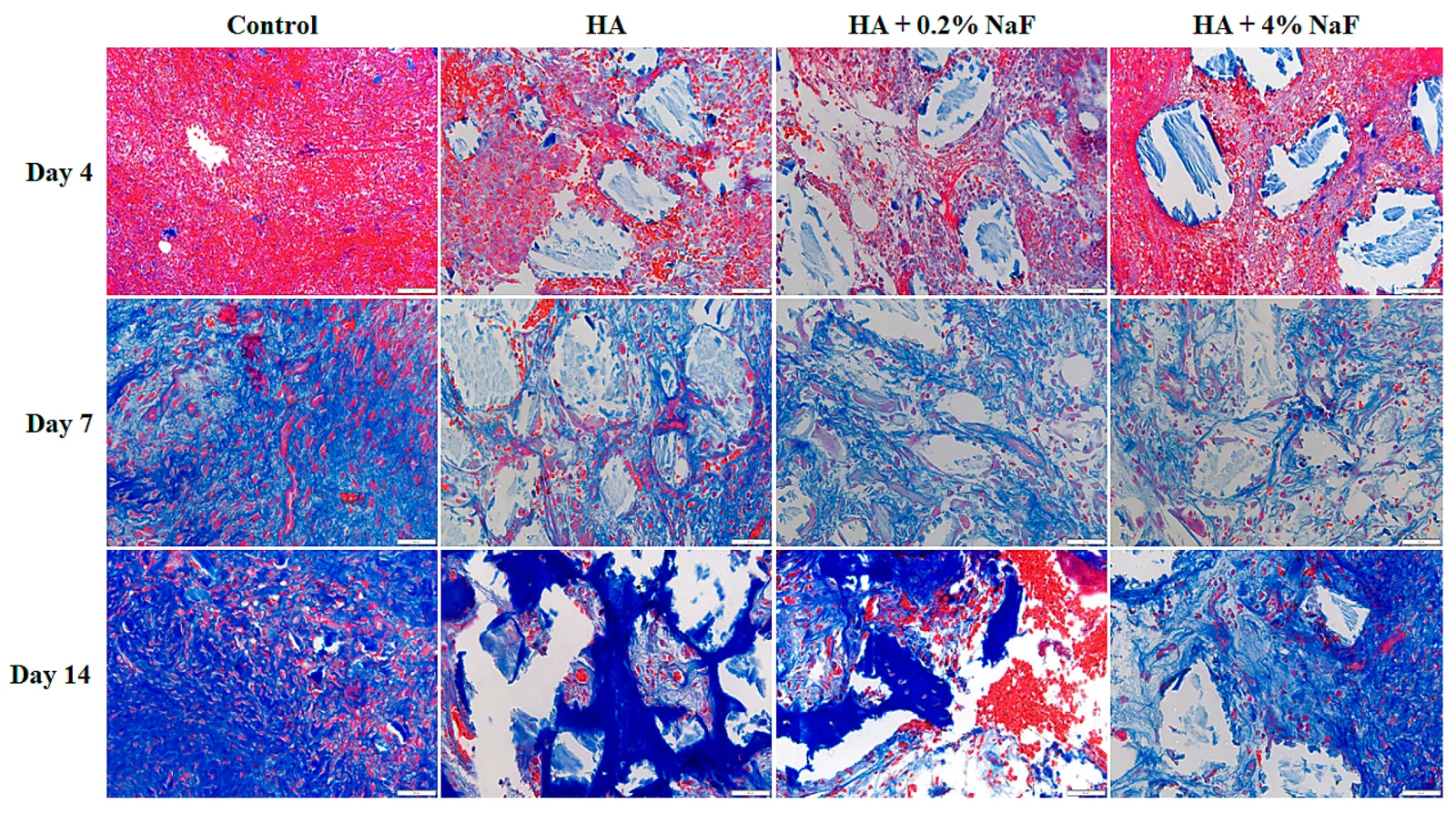
Histological evaluation of mandibular bone healing by Azan staining. Representative Azan-stained sections of the Control, HA, HA + low NaF (0.2%), and HA + high NaF (4%) groups at Days 4, 7, and 14. The sections show changes in tissue organization and bone-associated extracellular matrix formation around the implanted HA during healing. Original magnification, ×20; scale bar = 50 μm.

Histological changes were examined using Azan, Masson’s trichrome, toluidine blue, and H&E staining at Days 4, 7, and 14. At Day 4, the morphology and size of the implanted HA particles were largely maintained in the fluoride-treated groups, with residual HA surrounded by cellular and extracellular matrix components. Progressive tissue organization surrounding the implanted material was observed on days 7 and 14. In the HA + low NaF group, newly formed bone and osteoblast-like cells were more prominent around the HA at Days 7 and 14. Azan staining showed progressive organization of collagenous and mineralized tissue (Figure 1). Representative three-dimensional micro-CT reconstructions at Days 4, 7, and 14 are presented in Supplementary Figure S1.

**Figure 2:**
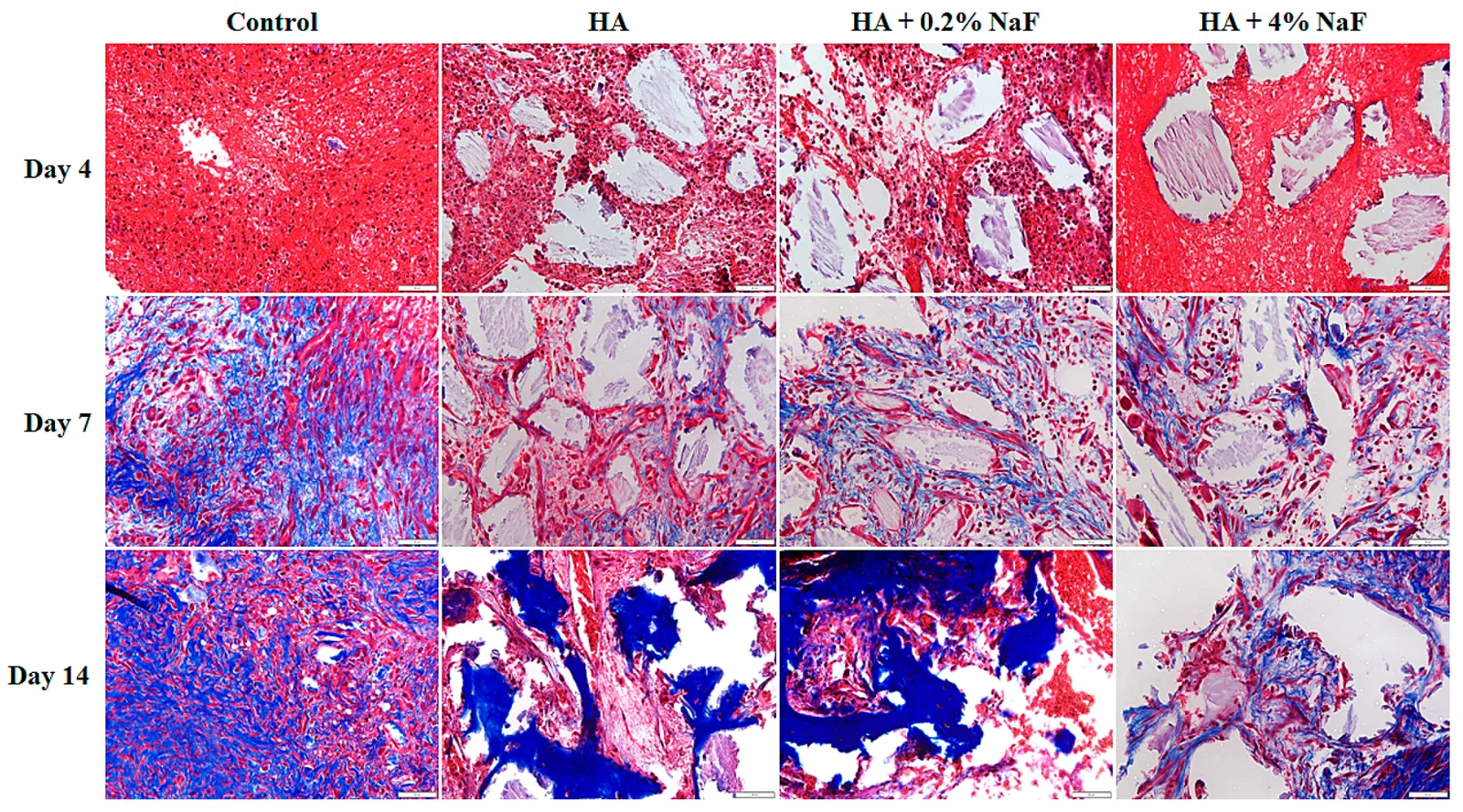
Histological evaluation of mandibular bone healing by Masson’s trichrome staining. Representative Masson’s trichrome-stained sections of the Control, HA, HA + low NaF (0.2%), and HA + high NaF (4%) groups at Days 4, 7, and 14. Changes in collagen-rich connective tissue and newly forming bone matrix around the implanted HA are shown. Original magnification, ×20; scale bar = 50 μm.

Masson’s trichrome stain showed progressive collagen-rich matrix around the implanted particles (Figure 2).

**Figure 3:**
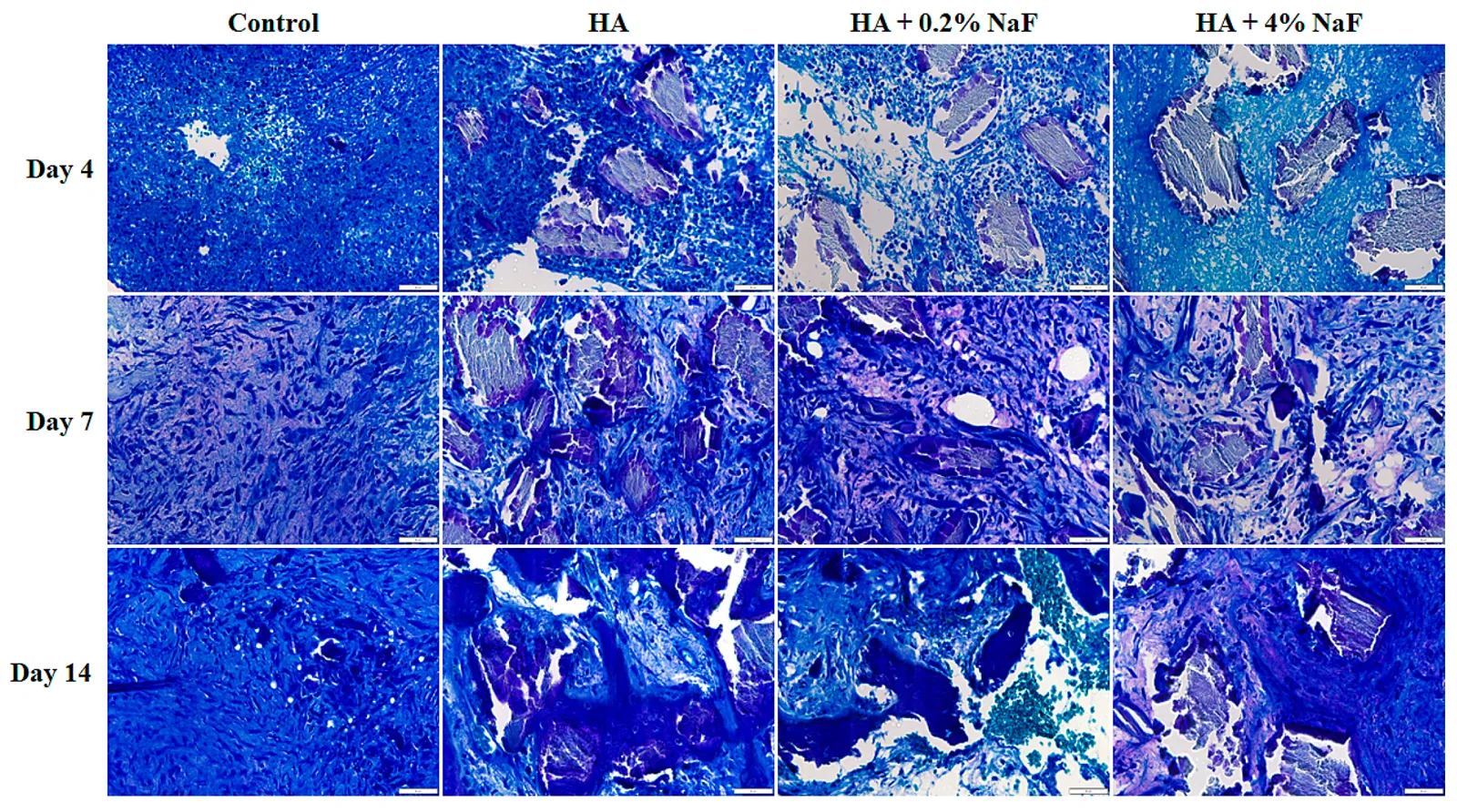
Histological evaluation of mandibular bone healing by toluidine blue staining. Representative toluidine blue-stained sections of the Control, HA, HA + low NaF (0.2%), and HA + high NaF (4%) groups at Days 4, 7, and 14. The sections show changes in cellular organization and extracellular matrix surrounding the implanted material during healing. Original magnification, ×20; scale bar = 50 μm.

Toluidine blue staining showed temporal variations in cellular and extracellular matrix arrangement (Figure 3).

**Figure 4:**
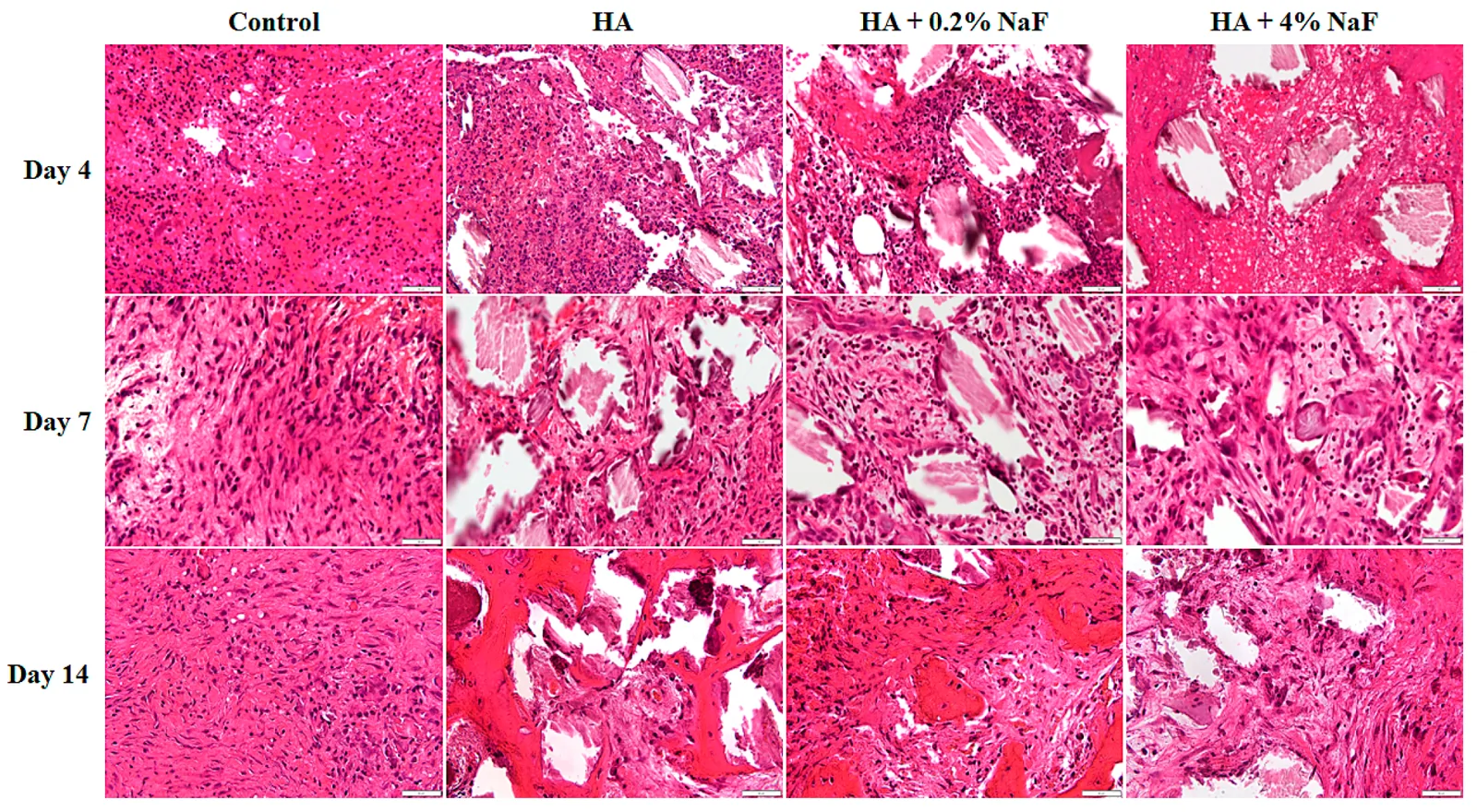
Histological evaluation of mandibular bone healing by hematoxylin and eosin staining. Representative H&E-stained sections of the Control, HA, HA + low NaF (0.2%), and HA + high NaF (4%) groups at Days 4, 7, and 14. Progressive changes in cellularity and tissue organization within the defect area and around the implanted HA are shown. Original magnification, ×20; scale bar = 50 μm.

H&E staining showed progressive changes in cellularity and tissue architecture during the observation period (Figure 4). Representative low-magnification H&E-stained images showing the overall morphology of the defect specimens in all experimental groups at Days 4, 7, and 14 are provided in Supplementary Figure S2.

### 2.2. Immunohistochemical Expression of Runx2

**Figure 5:**
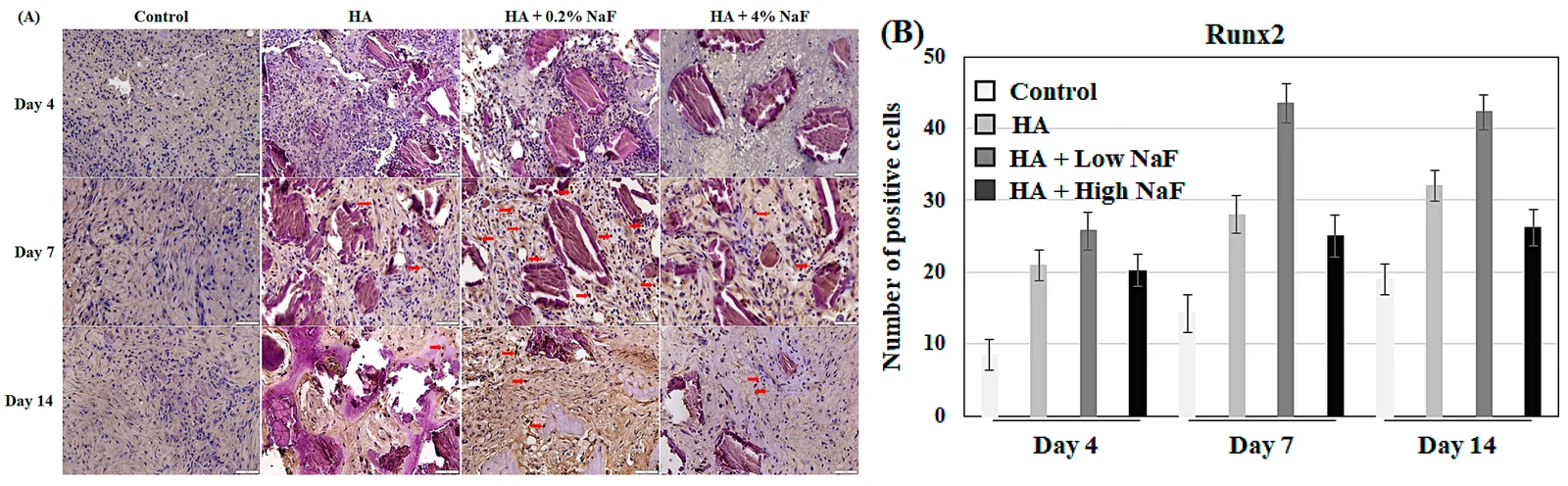
Runx2 expression during early mandibular bone healing. (**A**) Representative immunohistochemical staining for Runx2 in the Control, HA, HA + low NaF (0.2%), and HA + high NaF (4%) groups at Days 4, 7, and 14. Runx2-positive cells are identified by brown DAB staining, showing their distribution within the healing defect and around the implanted HA. Brown DAB staining indicates Runx2-positive cells and red arrows highlight representative areas of positive immunostaining.Original magnification, ×20; scale bar = 50 μm. (**B**) Quantitative evaluation of Runx2-positive cells. Values are presented as mean ± SD of four separately analyzed socket samples per condition. Each HA-based treatment condition at each time point was represented by one animal (*n* = 1); therefore, the four defect samples were considered within-animal subsamples rather than independent biological replicates, and the error bars reflect within-animal variability. All comparisons are descriptive and exploratory, without inferential statistical testing.

Runx2 immunohistochemistry showed temporal differences in the distribution of Runx2-positive cells among the experimental groups. Runx2 immunoreactivity was detected at the bone-substitute interface during the early healing period and became prominent in the HA + low NaF group. Positive cells were still observed at Day 14 with different staining intensity and distribution in the HA, HA + low NaF and HA + high NaF groups (Figure 5).

### 2.3. Immunohistochemical Expression of Osteocalcin

**Figure 6:**
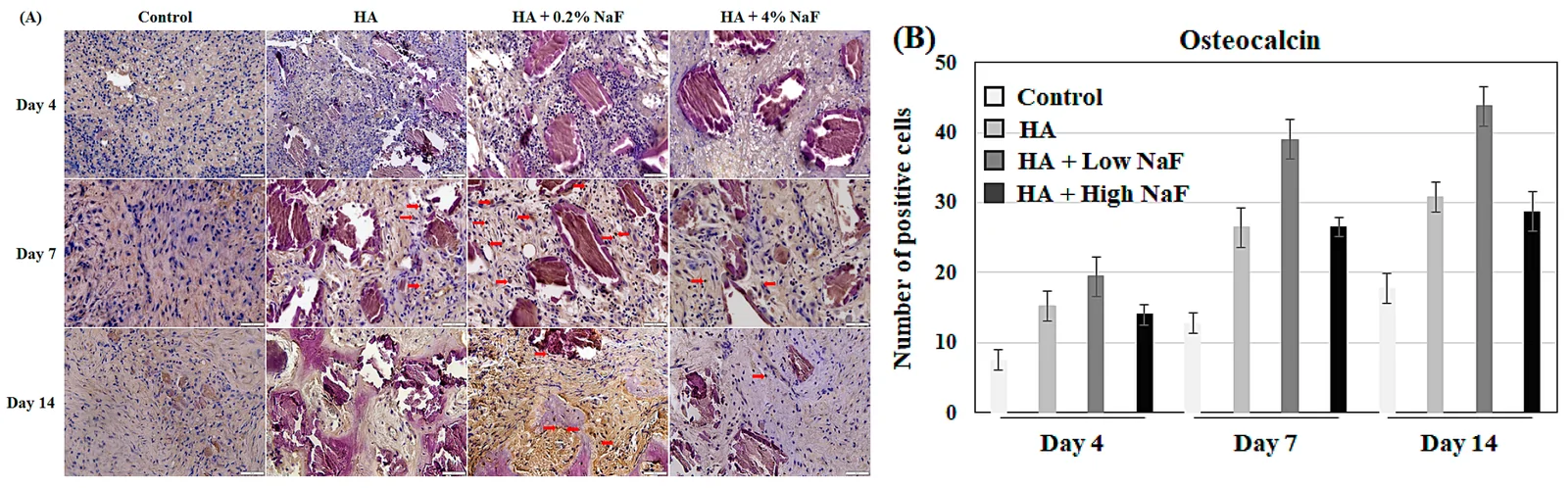
Osteocalcin expression during early mandibular bone healing. (**A**) Representative immunohistochemical staining for osteocalcin in the Control, HA, HA + low NaF (0.2%), and HA + high NaF (4%) groups at Days 4, 7, and 14. Osteocalcin-positive staining is shown within the healing tissue and around areas of newly forming bone. Brown DAB staining indicates osteocalcin-positive cells, and red arrows highlight representative areas of osteocalcin-positive immunostaining.Original magnification, ×20; scale bar = 50 μm. (**B**) Quantitative evaluation of osteocalcin-positive cells. Values are presented as mean ± SD of four separately analyzed socket samples per condition. Each HA-based treatment condition at each time point was represented by one animal (*n* = 1); therefore, the four defect samples were considered within-animal subsamples rather than independent biological replicates, and the error bars reflect within-animal variability. All comparisons are descriptive and exploratory, without inferential statistical testing.

Osteocalcin immunoreactivity was detected at the bone-substitute interface during healing and became more evident as the observation period progressed. In the HA + low NaF group, the staining was most prominent, especially in cells and tissue surrounding the implanted particles. Osteocalcin-positive cells were also observed in the HA and HA + high NaF groups, with differences in distribution and staining intensity (Figure 6).

### 2.4. Runx2 and Osteocalcin mRNA Expression

**Figure 7:**
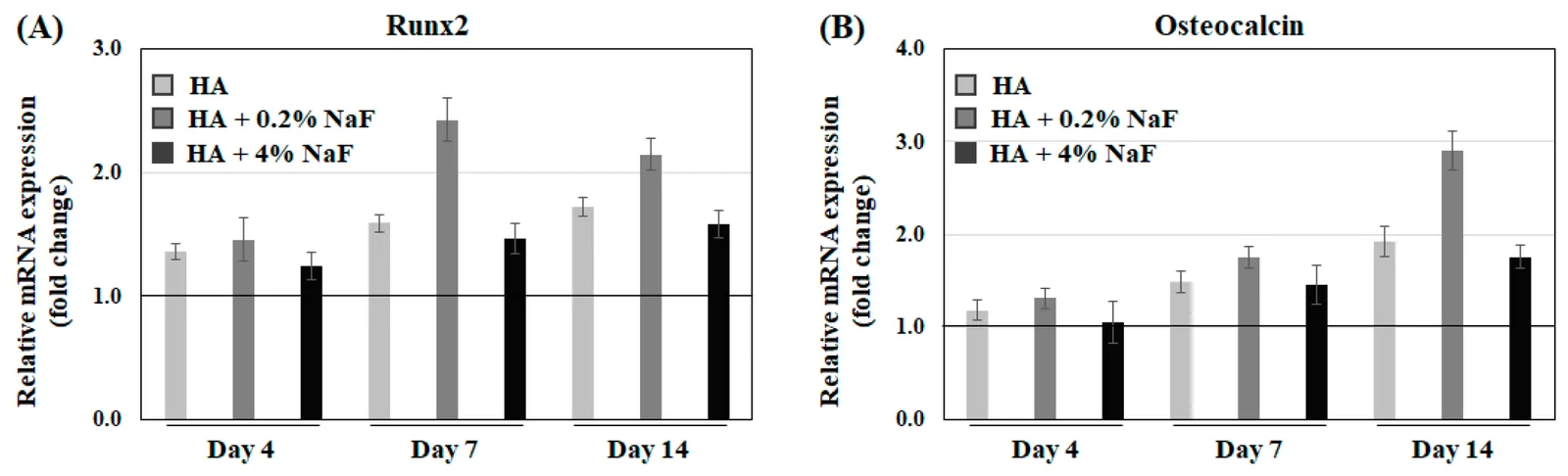
Relative mRNA expression of Runx2 and osteocalcin during early bone healing. Relative mRNA expression of (**A**) Runx2 and (**B**) osteocalcin at Days 4, 7, and 14. Gene expression was normalized to ACTB and calculated using the 2^−ΔΔCt^ method, with the mean Control ΔCt at each observation time point used as the calibrator (relative expression = 1.0, indicated by the horizontal reference line). Data for HA, HA + 0.2% NaF, and HA + 4% NaF are presented as mean ± SD of four separately analyzed within-animal socket samples. Each HA-based treatment condition at each time point was represented by one independent animal (*n* = 1); therefore, the four defect samples were considered within-animal subsamples rather than independent biological replicates, and the error bars reflect within-animal variability. All comparisons are descriptive and exploratory, without inferential statistical testing.

Descriptive qRT-PCR analysis showed variations in Runx2 expression among the treatment conditions and observation time points. At Day 4, Runx2 expression was higher in the HA-containing conditions than in the corresponding controls. The HA + low NaF condition showed the highest observed Runx2 expression at Days 7 and 14 (Figure 7A). These findings represent descriptive observations because each treatment at each time point was represented by one animal.

A similar descriptive pattern was observed for osteocalcin expression. At Day 4, osteocalcin expression was higher in the HA-containing conditions than in the corresponding controls. The HA + low NaF condition showed the highest observed osteocalcin expression at Days 7 and 14, with a more pronounced response at Day 14 (Figure 7B). These observations require confirmation in experiments with independent animal-level replication.

## 3. Discussion

The present study examined the early tissue and molecular responses to resorbable HA pretreated with low or high concentrations of NaF in a beagle mandibular defect model. The most notable finding was that the response was not simply greater with increasing fluoride concentration. HA pretreated with 0.2% NaF showed the strongest Runx2 and osteocalcin responses and was accompanied by more evident new bone and osteoblast-associated histological changes at Days 7 and 14. HA pretreated with 4% NaF also showed osteogenic activity, but the response was less pronounced compared to that with 0.2% NaF. These results indicate different early bone-healing responses between the two NaF pretreatment conditions.

This pattern is consistent with previous work on fluoride-modified apatites. Fluoride incorporation can alter the crystal and surface properties of HA, and earlier studies from this research line demonstrated both the preparation of crystalline fluorapatite-coated HA and osteogenesis around fluoridated HA in dogs [4,5]. Fluoride-treated bioresorbable synthetic HA has also shown regenerative potential in beagle mandibular defects [6]. Independent canine studies subsequently reported improved bone formation with fluorinated HA compared with non-fluorinated HA [7]. Similar findings have since been reported with fluorapatite and fluoride-substituted HA scaffolds, supporting the broader potential of fluoride-modified apatite materials for bone regeneration and dental applications [8,9,10,11,25,26,27]. The present findings add to this evidence by focusing specifically on the first two weeks of healing and linking the tissue response to the temporal expression of Runx2 and osteocalcin.

Previous studies using the same resorbable HA material demonstrated that treatment with approximately 4% NaF can modify the HA surface and result in detectable surface fluoride, as assessed by X-ray diffraction, EDX analysis, and elemental mapping [4]. Moreover, fluoride release from the same HA material pretreated with 4% NaF for 3 min has been demonstrated in vitro, with an initial rapid release followed by slower release over 48 h [28]. However, these previous findings were obtained under their respective experimental conditions and should not be considered direct physicochemical characterization of the specimens used in the present study. Fluoride uptake, retention, and release were not directly quantified for the 0.2% and 4% NaF-pretreated HA specimens used here. Accordingly, the actual fluoride content of the implanted HA, its retention after washing and sterilization, and its subsequent release into the local tissue environment remain unknown. This prevents direct correlation of the observed biological responses with the amount of fluoride incorporated into or released from the implanted material.

The more pronounced osteogenic response observed with HA pretreated with 0.2% NaF is also in line with the well-recognized concentration-dependent biology of fluoride [12,13]. Micromolar NaF was found to enhance osteoblast proliferation, alkaline phosphatase activity, mineralization, and expression of several osteogenic markers, including Runx2 and osteocalcin [14]. In our previous study, low-level NaF promoted proliferation and migration of bone marrow mesenchymal stem cells and enhanced mineralization together with Runx2 and osteocalcin expression [15]. Other work from our group has shown that low fluoride concentrations can stimulate epithelial-cell migration and epithelial–mesenchymal interactions relevant to tissue repair [16,17]. Together, these studies support the idea that controlled low-level fluoride exposure can create conditions that favor wound healing and osteogenic differentiation. This interpretation is also supported by recent studies of fluorinated HA, in which fluoride incorporation enhanced osteogenic responses while favorably modulating the local regenerative microenvironment [19,20]. More recently, fluorine-incorporated biogenic HA was shown to enhance new bone formation and mineralization during alveolar socket healing, together with reduced macrophage-mediated inflammation and enhanced osteogenic differentiation [29].

The temporal expression pattern observed here is consistent with the progression of early osteogenic differentiation. Descriptively, Runx2 expression was highest at Day 7, whereas osteocalcin expression showed its highest levels at Day 14, consistent with the expected transition from early osteogenic differentiation toward matrix maturation. Runx2 is important for commitment to the osteoblast lineage and early differentiation [22,23,24]. Osteocalcin is associated with a more differentiated osteoblast phenotype and mineralizing matrix. Thus, the early Runx2 response and the later osteocalcin response fit with the expected sequence of osteogenic differentiation during healing.

The histological findings paralleled these molecular changes. H&E, Azan, Masson’s trichrome and toluidine blue staining revealed progressive organization of the cells and extracellular matrix around the implanted HA. On Days 7 and 14, new bone and osteoblast-like cells were more pronounced around low-NaF-pretreated HA. Importantly, the HA was exposed to NaF for only 3 min and then washed with distilled water before implantation. Thus, this model reflects the biological response to fluoride-pretreated HA rather than direct application of the original NaF solution to the defect.

In this early-stage mandibular defect model, changes in osteogenesis-related molecular markers may become detectable before substantial morphological differences are evident. The relatively subtle histological differences between the treatment conditions during the first 14 days may therefore reflect the early stage of tissue remodeling, although this interpretation requires confirmation through quantitative histomorphometry and studies with independent animal-level replication.

The results in the 4% NaF group should be interpreted with caution as well. They do not indicate complete inhibition of osteogenic activity, because Runx2 and osteocalcin expression remained detectable. Instead, the higher pretreatment concentration did not offer the extra advantage observed with 0.2% NaF. This difference is consistent with the biphasic effects of fluoride [12,13]. High-concentration NaF exposure has been related to oxidative stress, apoptosis, dysregulated autophagy, and periodontal tissue injury [18]. Lower concentrations, however, can induce vascularized bone formation [20,30] and inhibit osteoclastogenesis and age-related alveolar bone resorption [31]. The balance between these responses may help explain why more fluoride did not produce a stronger regenerative effect in the present model. More broadly, fluoride incorporation into other bioactive bone-substitute materials has also been associated with enhanced osteogenic responses in vitro and in vivo [32].

Several signaling pathways may contribute to the different responses observed between the two NaF pretreatment conditions, although they were not examined directly in the present study. Fluoride has been reported to promote osteoblast differentiation through canonical Wnt/β-catenin signaling [33], and dose-dependent changes in BMP-2 and BMP-3 expression, proliferation, and apoptosis have also been described in osteoblast-like cells [34]. These observations provide a plausible mechanistic context for the enhanced Runx2 response seen with low-NaF-pretreated HA, but direct pathway analysis will be needed before a specific mechanism can be assigned.

A strength of this study is the use of a large-animal mandibular defect model with three early observation points, allowing the temporal patterns of Runx2 and osteocalcin expression to be examined within the same experimental framework. However, several limitations should be acknowledged. First, each treatment condition at each observation time point was represented by only one animal. Although four defect samples were obtained from each animal, these samples were not independent biological replicates because all four experimental defects received the same treatment. Consequently, the four defect measurements reflect within-animal variability rather than independent biological replication, and no conclusions regarding statistically significant treatment effects can be drawn. All observed differences between treatment conditions and observation time points should therefore be interpreted as descriptive and exploratory findings requiring confirmation in independently replicated animal experiments. In addition, treatment and control sides were predetermined rather than randomized; therefore, potential side- or anatomical-site-related effects cannot be completely excluded. Second, the observation period ended at Day 14, leaving uncertainty about whether the early responses persist during later bone maturation and remodeling. Third, quantitative histomorphometry and quantitative micro-CT parameters were not included. Furthermore, although previous studies using the same HA material under related NaF-treatment conditions demonstrated surface fluoride and subsequent fluoride release [4,28], fluoride uptake, retention, and release were not directly measured in the specimens used in the present study, and physicochemical characterization by XRD, EDX, or FTIR was not performed. The signaling pathways underlying the observed responses were also not investigated directly. Future studies incorporating independent animal-level replication, randomized treatment allocation, longer observation periods, quantitative bone measurements, and material characterization are needed to determine whether low-concentration NaF pretreatment improves long-term bone regeneration.

## 4. Materials and Methods

### 4.1. Animals and Experimental Design

Twelve healthy female TOYO beagle dogs, approximately 24 months of age and weighing 10.6 ± 1.0 kg, were used in this project. Animals were housed individually under standard laboratory conditions throughout the experimental period, with regular food and water and a standard temperature and light cycle. Three dogs were used in a pilot study to establish the surgical procedures and treatment allocation and were not included in the main experimental analysis. The remaining nine dogs were included in the main experiment, with three dogs assigned to each observation time point (Days 4, 7, and 14). At each time point, one dog was assigned to each of the three HA-based treatments: untreated HA, HA pretreated with 0.2% NaF (HA + low NaF), or HA pretreated with 4% NaF (HA + high NaF). All four experimental defects within each dog received the same assigned treatment, while four contralateral defects were left unfilled as controls. Tissue specimens were collected for histological examination, immunohistochemical analysis and quantitative real-time reverse transcription-polymerase chain reaction (RT-PCR). All experimental procedures were reviewed and approved by the Animal Safety and Ethics Committee of Kanagawa Dental University (Approval Nos. 17-027, 17-034, and 17-035).

### 4.2. Surgical Procedure and Preparation of Fluoride-Treated HA

For surgical procedures, the animals were sedated by intramuscular administration of medetomidine hydrochloride (Domitor, Nippon Zenyaku Kogyo Co., Ltd., Koriyama, Japan; 0.5–1.0 mL), followed by intravenous administration of sodium pentobarbital (Somnopentyl, Kyoritsu Seiyaku Corporation, Chiyoda-ku, Japan; 3.0–4.0 mL) for general anesthesia. Local infiltration anesthesia was administered before and during the surgical procedure at the oral surgical site using a dental Xylocaine cartridge (Dentsply Sirona, Chuo City, Japan). For perioperative analgesia, Vetorphale 5 mg (Meiji Seika Pharma Co., Ltd., Chuo-ku, Japan) was administered intramuscularly at 0.04–0.06 mL/kg. No antibiotics were administered.

The bilateral mandibular posterior teeth were extracted, and the extraction sites were allowed to heal for 18–20 weeks before preparation of the experimental defects. Under general anesthesia, the mandibular bone was surgically exposed, and four standardized cylindrical osseous defects (3.3 mm in diameter × 7.0 mm in depth) were prepared. Treatment allocation was predetermined rather than randomized. Four standardized defects were prepared on each mandibular side. All four defects on the experimental side received the same assigned HA-based treatment, whereas the four contralateral defects were left unfilled as controls. A schematic representation of the experimental design and defect allocation, together with representative photographs of the surgical procedures, treatment allocation, wound closure, and specimen collection, is provided in Supplementary Figure S3.

OsteoGen^®^ HA Resorb^®^ (Impladent Ltd., Holliswood, NY, USA), a resorbable calcium apatite-based bone graft material composed of hydroxyapatite crystals (300–400 μm), was used in this study. The material was sterilized using hydrogen peroxide gas plasma sterilization.

The NaF concentrations were selected based on practical considerations related to fluoride use in dental care and the preparation of aqueous NaF solutions. The lower concentration (0.2% NaF; approximately 900 ppm fluoride) was selected as a low-concentration pretreatment condition, whereas 4% NaF was used to examine the response to a substantially higher concentration. A short immersion period of 3 min was adopted as a standardized surface pretreatment procedure. The NaF solutions were used exclusively for HA pretreatment and were not applied directly to the bone defects.

For fluoride pretreatment, the HA was exposed to either 0.2% NaF (low NaF) or 4% NaF (high NaF) solution for 3 min, washed with 100 mL of distilled water, air-dried, and sterilized by hydrogen peroxide gas plasma before implantation. Untreated HA was used for the HA-only group. The untreated or NaF-pretreated HA was then placed into the designated defects.

The surgical sites were closed, and the animals were maintained postoperatively according to the established protocol. At the designated observation time points (Days 4, 7, and 14), the animals were sedated by intramuscular administration of medetomidine hydrochloride (Domitor; 1.0 mL), followed by intravenous administration of sodium pentobarbital (Somnopentyl; 6.0 mL) for euthanasia. Following euthanasia, specimens containing the implanted material and surrounding tissue within the experimental defects were collected using a 3.3 mm-diameter dental trephine bur. Corresponding tissue specimens were obtained from the contralateral non-implanted defects and used as controls. Each trephine specimen was physically divided into two portions. One portion was processed for decalcified histological examination and immunohistochemistry, while the other was used for RNA extraction and subsequent qRT-PCR analysis. Sampling was confined to tissue within the experimental defects, without inclusion of surrounding native bone. Animals were monitored throughout the experimental period according to the approved institutional protocol, and no unexpected adverse events were observed.

Micro-computed tomography (micro-CT) imaging was performed on harvested mandibular bone specimens using a three-dimensional X-ray computed tomography system (TDM3001-FP; Yamato Scientific Co., Ltd., Chuo-ku, Japan). Specimens collected at Days 4, 7, and 14 were examined to visualize the morphology of the experimental defect regions. Three-dimensional reconstructions were used for qualitative assessment only. Quantitative micro-CT parameters, including bone volume fraction and bone mineral density, were not evaluated, and no inferential comparisons were performed.

### 4.3. Histological Analysis

Mandibular tissue specimens were fixed, decalcified, dehydrated, embedded in paraffin, and sectioned at a thickness of 4 μm. Serial sections were stained with hematoxylin and eosin (H&E), Azan, Masson’s trichrome and toluidine blue to assess cellular and extracellular matrix changes, tissue organization and new bone formation at the experimental sites. Azan staining was performed using reagents from Muto Pure Chemicals (Tokyo, Japan); Masson’s trichrome staining was performed using ab150686 (Abcam, Tokyo, Japan), and toluidine blue staining was performed using reagents from Wako (Tokyo, Japan). Images were taken using an OLYMPUS microscope (Olympus Corporation, Tokyo, Japan). For comparative evaluation, high-magnification images were acquired from corresponding anatomical regions of the experimental defects using a consistent region-of-interest selection approach across all experimental groups and observation time points. Corresponding regions were selected from serial sections used for the different histological stains and immunohistochemical analyses, allowing direct comparison of histological features and immunohistochemical findings.

### 4.4. RNA Extraction and cDNA Synthesis

Total RNA was extracted from the tissue specimens using the miRNeasy Mini Kit (Qiagen K.K., Tokyo, Japan) according to the manufacturer’s protocol. The quantity and quality of isolated RNA were checked by an Agilent Bioanalyzer (Agilent Technologies, Santa Clara, CA, USA). For mRNA analysis, 1 µg of total RNA was reverse transcribed to cDNA using the High-Capacity cDNA Archive Kit (Thermo Fisher Scientific, Waltham, MA, USA) according to the manufacturer’s instructions.

### 4.5. Quantitative Real-Time RT-PCR

Quantitative real-time RT-PCR was performed using TaqMan Gene Expression Assays (Thermo Fisher Scientific, Waltham, MA, USA) according to the manufacturer’s protocol. The mRNA expression levels of Runx2 and osteocalcin (Bglap) were assessed as markers of osteogenic differentiation. The TaqMan assays used were Runx2 (Cf02694692_m1) and Bglap/osteocalcin (Cf02623891_g1), with ACTB (Cf04931159_m1) serving as the endogenous reference gene. Relative gene expression was calculated using the comparative Ct (2^−ΔΔCt^) method, with the mean ΔCt of the corresponding Control samples at each observation time point as the calibrator (relative expression = 1.0). Data are presented descriptively as mean ± SD of four separately analyzed within-animal socket samples, and no inferential statistical comparisons between treatment groups or observation time points were performed.

### 4.6. Immunohistochemistry

Immunohistochemical analysis was performed on 4-μm paraffin-embedded mandibular tissue sections. Sections were deparaffinized and rehydrated, followed by antigen retrieval using 10× Citrate Buffer, pH 6.0 (Abcam, Tokyo, Japan) at 95 °C for 12 min. Endogenous peroxidase activity was blocked before incubation with the primary antibodies. Anti-Runx2 antibody (Abcam, Tokyo, Japan) was used at 1:100, and anti-osteocalcin antibody (Abcam, Tokyo, Japan) was used at 1:75. Following incubation with the primary antibodies, sections were incubated with the appropriate secondary antibody. Immunoreactivity was visualized using 3,3′ diaminobenzidine (DAB). Isotype-matched control IgG was used as a negative control. Specimens from all experimental groups were immunostained simultaneously to ensure equivalent antibody-reaction and DAB-exposure conditions. Images were captured using an OLYMPUS microscope (Tokyo, Japan). High-magnification immunohistochemical images were acquired from corresponding anatomical regions in serial sections, consistent with the regions evaluated by histological staining, to facilitate direct comparison of histological and immunohistochemical findings across experimental groups and observation time points. Runx2- and osteocalcin-positive cells were identified by brown DAB staining and counted in four microscopic fields per defect sample. The counts from the four microscopic fields were averaged to obtain a mean positive-cell count per field for each defect sample. These defect-level mean values were summarized as mean ± SD across the four defect samples obtained from each animal, reflecting within-animal variability rather than independent biological replication. Outcome assessment for the histological, immunohistochemical, and molecular analyses was performed with investigators blinded to the experimental groups.

### 4.7. Statistical Analysis

Quantitative measurements were obtained separately from four defect samples per animal and summarized descriptively. Each HA-based treatment at each observation time point was represented by one animal. Because the four samples from each animal received the same treatment, they were considered within-animal observations rather than independent biological replicates. Accordingly, no inferential statistical comparisons between treatment groups were performed.

## 5. Conclusions

In this exploratory beagle mandibular defect study, HA pretreated with 0.2% NaF showed histological and molecular findings suggestive of a favorable early osteogenic response. The observations suggest that NaF pretreatment concentration may influence HA-associated bone healing. However, the limited animal-level replication prevents definitive conclusions regarding differences between treatment conditions. Further studies with independent biological replication and quantitative bone-healing assessments are required.

## Data Availability

The original contributions presented in this study are included in the article/Supplementary Material. Further inquiries can be directed to the corresponding author.

## Supplementary Materials

The following supporting information can be downloaded at: https://www.mdpi.com/article/10.3390/ijms27219414/s1.

## References

[1] Fendi F., Abdullah B., Suryani S., Usman A.N., Tahir D. (2024). Development and Application of Hydroxyapatite-Based Scaffolds for Bone Tissue Regeneration: A Systematic Literature Review. Bone.

[2] Ratnayake J.T.B., Mucalo M., Dias G.J. (2017). Substituted Hydroxyapatites for Bone Regeneration: A Review of Current Trends. J. Biomed. Mater. Res. Part B Appl. Biomater..

[3] Haider A., Haider S., Han S.S., Kang I.K. (2017). Recent Advances in the Synthesis, Functionalization and Biomedical Applications of Hydroxyapatite: A Review. RSC Adv..

[4] Kimoto K., Okudera T., Okudera H., Nordquist W.D., Krutchkoff D.J. (2011). Part I: Crystalline Fluorapatite-Coated Hydroxyapatite, Physical Properties. J. Oral Implantol..

[5] Nordquist W.D., Okudera H., Kitamura Y., Kimoto K., Okudera T., Krutchkoff D.J. (2011). Part II: Crystalline Fluorapatite-Coated Hydroxyapatite Implant Material: A Dog Study With Histologic Comparison of Osteogenesis Seen With FA-Coated HA Grafting Material Versus HA Controls: Potential Bacteriostatic Effect of Fluoridated HA. J. Oral Implantol..

[6] Masuki H., Kawabata H., Isobe K., Kimoto K., Okudera T., Okudera H. (2012). Analysis of jaw bone regeneration in a beagle dog with implantation of fluoride-treated bioresorbable synthetic hydroxyapatite. J. Jpn. Soc. Oral Implantol..

[7] Liu Y., Liu C., Huang R., Chen K., Huang B., Liu Q., Chen Z., Li Z. (2020). Effects of Fluorinated Porcine Hydroxyapatite on Lateral Ridge Augmentation: An Experimental Study in the Canine Mandible. Am. J. Transl. Res..

[8] Ratnayake J., Gould M., Ramesh N., Mucalo M., Dias G.J. (2024). A Porous Fluoride-Substituted Bovine-Derived Hydroxyapatite Scaffold Constructed for Applications in Bone Tissue Regeneration. Materials.

[9] Kazimierczak P., Wessely-Szponder J., Palka K., Barylyak A., Zinchenko V., Przekora A. (2023). Hydroxyapatite or Fluorapatite—Which Bioceramic Is Better as a Base for the Production of Bone Scaffold?—A Comprehensive Comparative Study. Int. J. Mol. Sci..

[10] Nielson C., Agarwal J., Beck J.P., Shea J., Jeyapalina S. (2024). Sintered fluorapatite scaffolds as an autograft-like engineered bone graft. J. Biomed. Mater. Res. B Appl. Biomater..

[11] He S., Hu Q., Sun Y., Xu Y., Huang L., Cao G., Guo T. (2023). Electrospun polycaprolactone incorporated with fluorapatite nanoparticles composite scaffolds enhance healing of experimental calvarial defect on rats. Ann. Transl. Med..

[12] Wang X., Tewari N., Sato F., Tanimoto K., Thangavelu L., Makishima M., Bhawal U.K. (2022). Biphasic Functions of Sodium Fluoride (NaF) in Soft and in Hard Periodontal Tissues. Int. J. Mol. Sci..

[13] Pak C.Y.C., Zerwekh J.E., Antich P. (1995). Anabolic Effects of Fluoride on Bone. Trends Endocrinol. Metab..

[14] Lee M., Arikawa K., Nagahama F. (2017). Micromolar Levels of Sodium Fluoride Promote Osteoblast Differentiation Through Runx2 Signaling. Biol. Trace Elem. Res..

[15] Bhawal U.K., Li X., Suzuki M., Taguchi C., Oka S., Arikawa K., Tewari N., Liu Y. (2020). Treatment with Low-level Sodium Fluoride on Wound Healing and the Osteogenic Differentiation of Bone Marrow Mesenchymal Stem Cells. Dent. Traumatol..

[16] Arakawa Y., Bhawal U.K., Ikoma T., Kimoto K., Kuroha K., Kubota T., Hamada N., Kubota E., Arakawa H. (2009). Low Concentration Fluoride Stimulates Cell Motility of Epithelial Cells in Vitro. Biomed. Res..

[17] He D., Bhawal U.K., Hamada N., Kuboyama N., Abiko Y., Arakawa H. (2013). Low Level Fluoride Stimulates Epithelial-Mesenchymal Interaction in Oral Mucosa. J. Hard Tissue Biol..

[18] Oka S., Li X., Zhang F., Taguchi C., Tewari N., Kim I.-S., Zhong L., Arikawa K., Liu Y., Bhawal U.K. (2020). Oral Toxicity to High Level Sodium Fluoride Causes Impairment of Autophagy. J. Physiol. Pharmacol..

[19] Chen K., Ha S., Xu L., Liu C., Liu Y., Wu X., Li Z., Wu S., Yang B., Chen Z. (2024). Fluorinated hydroxyapatite conditions a favorable osteo-immune microenvironment via triggering metabolic shift from glycolysis to oxidative phosphorylation. J. Transl. Med..

[20] Wu X., Xu C., Feng J., Wu S., Liu R., Qiao W., Luo X., Chen S., Li Z., Chen Z. (2024). Fluorinated Porcine Bone-Derived Hydroxyapatite Promotes Vascularized Osteogenesis by Coordinating Human Bone Marrow Mesenchymal Stem Cell/Human Umbilical Vein Endothelial Cell Complexes. Bioengineering.

[21] Shang L., Shao J., Ge S. (2022). Immunomodulatory Properties: The Accelerant of Hydroxyapatite-Based Materials for Bone Regeneration. Tissue Eng. Part C Methods.

[22] Komori T. (2010). Regulation of Osteoblast Differentiation by Runx2. Osteoimmunology.

[23] Vimalraj S., Arumugam B., Miranda P.J., Selvamurugan N. (2015). Runx2: Structure, Function, and Phosphorylation in Osteoblast Differentiation. Int. J. Biol. Macromol..

[24] Komori T. (2022). Whole Aspect of Runx2 Functions in Skeletal Development. Int. J. Mol. Sci..

[25] Seyedmajidi S., Seyedmajidi M., Haghanifar S. (2023). Optimization of Fluorapatite/Bioactive Glass Nanocomposite Foams as Bone Tissue Scaffold: An in Vivo Study. Int. J. Mol. Cell Med..

[26] Mirjalili F., Mahmoodi M., Khazali S. (2024). Characterization and in vitro bioactivity evaluation of polyvinyl alcohol incorporated electrospun chitosan/fluorapatite nanofibrous scaffold for bone tissue engineering. J. Mech. Behav. Biomed. Mater..

[27] Steyl S.K., Jeyapalina S., Griffin A., Krishnamoorthi V., Beck J.P., Agarwal J., Shea J. (2024). Efficacy of sintered Zinc-doped fluorapatite scaffold as an antimicrobial regenerative bone filler for dental applications. J. Dent..

[28] Ohno M., Kimoto K., Toyoda T., Kawata K., Arakawa H. (2013). Fluoride-Treated Bio-Resorbable Synthetic Hydroxyapatite Promotes Proliferation and Differentiation of Human Osteoblastic MG-63 Cells. J. Oral Implantol..

[29] Liu C., Xu L., Feng J., Yang B., Chen K., Liu Y., Wu X., Wu S., Li Z., Chen S. (2025). Fluorine-Incorporated Biogenic Hydroxyapatite Enhances Socket Bone Healing via Addressing Macrophage-Mediated Inflammatory Response. Bioengineering.

[30] Wu S., Xia B., Mai S., Feng Z., Wang X., Liu Y., Liu R., Li Z., Xiao Y., Chen Z. (2019). Sodium Fluoride under Dose Range of 2.4-24 ΜM, a Promising Osteoimmunomodulatory Agent for Vascularized Bone Formation. ACS Biomater. Sci. Eng..

[31] Oka S., Li X., Taguchi C., Wang C., Tewari N., Arikawa K., Liu Y., Bhawal U.K. (2021). Treatment with 50 ΜM Sodium Fluoride Suppresses Aging-Induced Alveolar Bone Resorption in Mice. J. Hard Tissue Biol..

[32] Chen X., Liu Y., Zhao Y., Ouyang Z., Zhou H., Li L., Li L., Li F., Xie X., Hill R.G. (2022). Halide-containing bioactive glasses enhance osteogenesis in vitro and in vivo. Biomater. Adv..

[33] Pan L., Shi X., Liu S., Guo X., Zhao M., Cai R., Sun G. (2014). Fluoride Promotes Osteoblastic Differentiation through Canonical Wnt/β-Catenin Signaling Pathway. Toxicol. Lett..

[34] Wei Y., Wu Y., Zeng B., Zhang H. (2014). Effects of Sodium Fluoride Treatment In Vitro on Cell Proliferation, BMP-2 and BMP-3 Expression in Human Osteosarcoma MG-63 Cells. Biol. Trace Elem. Res..

