# Supplementary material for: Effects of Hydroxyapatite Pretreated with Two Sodium Fluoride Concentrations on Early Bone Healing in Beagle Dogs
Source: Int J Mol Sci. 2026 Oct 22;27(21):9414. doi: 10.3390/ijms27219414 (PMC13762727; doi:10.3390/ijms27219414)
Supplement: Supplementary file 1 [file ijms-27-09414-s001.zip › Supplementary Figure Legends (Round 2).pdf]

Supplementary Figure S1. Representative three-dimensional micro-CT reconstructions of the mandibular defect specimens during early bone healing. Representative three-dimensional reconstructions of harvested mandibular bone specimens from the Control, HA, HA + 0.2% NaF, and HA + 4% NaF groups at Days 4, 7, and 14. Micro-CT imaging was performed using a three-dimensional X-ray computed tomography system (TDM3001-FP; Yamato Scientific Co., Ltd., Japan), which has a specified tube voltage range of 30–300 kV and a minimum pixel resolution of 10 μm. The reconstructions are presented for qualitative visualization of the experimental defect regions and were not used for quantitative micro-CT analysis.

Supplementary Figure S2. Low-magnification histological overview of the experimental bone defects. Representative H&E-stained sections showing the overall morphology of the defect specimens in the Control, HA, HA + 0.2% NaF, and HA + 4% NaF groups at Days 4, 7, and 14. Images were acquired using a ×2 objective. Scale bar = 500 μm.

Supplementary Figure S3. Experimental design, mandibular defect allocation, and surgical procedure. (a) Schematic representation of the experimental design and defect allocation. Twelve beagle dogs were included in the overall project; three were used for preliminary procedures and nine were included in the main experiment. Three animals were examined at each observation time point (Days 4, 7, and 14), with one animal assigned to each HA-based treatment condition (HA, HA + 0.2% NaF, or HA + 4% NaF). In each animal, four standardized defects on one mandibular side received the same assigned HA-based treatment, while four corresponding defects on the contralateral side were left unfilled as controls. Treatment and control sides were predetermined rather than randomized. (b) Representative photographs of surgical procedures and specimen collection. (A) Mandibular premolars before extraction. (B) Extraction sockets following premolar removal. (C) Healed extraction sites before defect preparation. (D) Preparation of standardized mandibular bone defects. (E) Four experimental defects assigned to the same HA-based treatment (HA, HA + 0.2% NaF, or HA + 4% NaF). (F) HA-based treatment sites following material placement. (G) Four unfilled control defects prepared on the contralateral mandibular side. (H) Wound closure following the surgical procedure. (I) Representative bone specimen collected for subsequent analyses.
